# Supplementary material for: Independent expansion, selection and hypervariability of the TBC1D3 gene family in humans
Source: bioRxiv. 2024 Mar 13:2024.03.12.584650. Preprint. [Version 1] doi: 10.1101/2024.03.12.584650 (PMC11037872; doi:10.1101/2024.03.12.584650)
Supplement: 2 [file NIHPP2024.03.12.584650v1-supplement-2.pdf]

bioRxiv preprint doi: https://doi.org/10.1101/2024.03.12.584650; this version posted March 13, 2024. The copyright holder for this preprint (which was not certified by peer review) is the author/funder, who has granted bioRxiv a license to display the preprint in perpetuity. It is made available under aCC-BY 4.0 International license.

SUPPLEMENTARY FIGURES

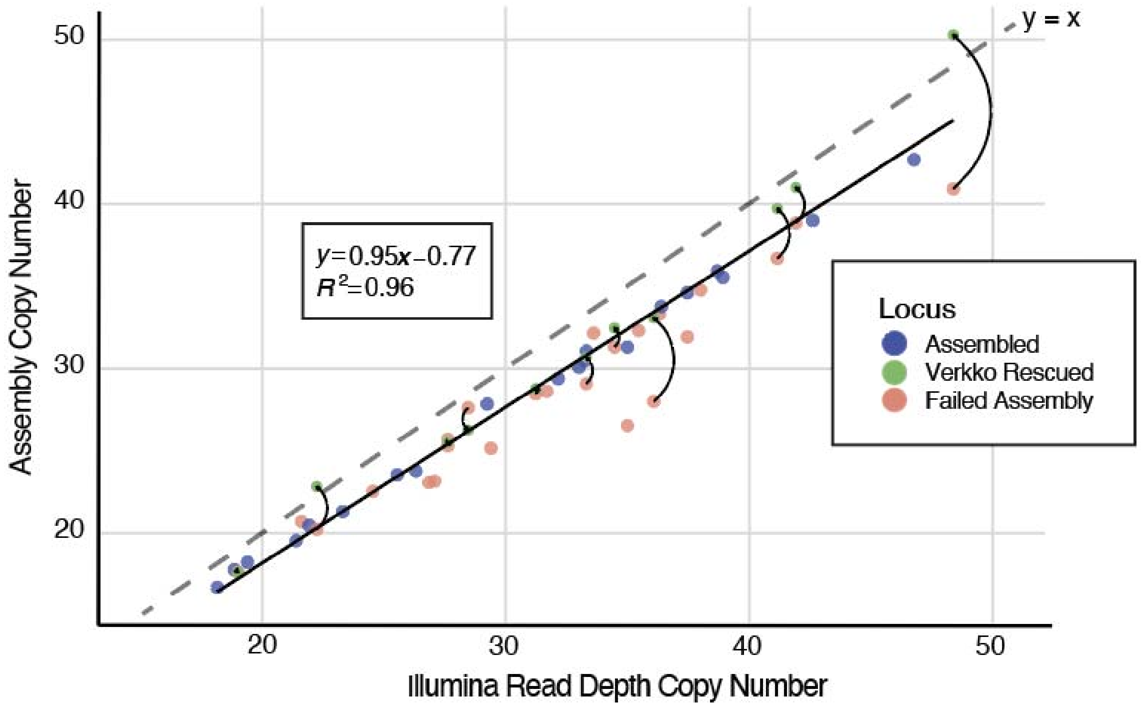

Supplementary Figure S1: Assembly and rescue of *TBC1D3* phased haplotypes. Samples were first phased and assembled using exclusively HiFi sequence. The y-axis represents the diploid copy number of these assemblies, and the x-axis represents diploid copy number based on Illumina sequence, with y=x axis marked with a dashed line. Assemblies were validated by read depth estimates of HiFi and ONT, colored in blue if validated, and salmon colored if failing validation. We attempted to rescue sample haplotypes using a novel assembly approach leveraging both HiFi and ultra-long ONT. These samples, assembled by Verkko, are indicated in green.

bioRxiv preprint doi: https://doi.org/10.1101/2024.03.12.584650; this version posted March 13, 2024. The copyright holder for this preprint (which was not certified by peer review) is the author/funder, who has granted bioRxiv a license to display the preprint in perpetuity. It is made available under aCC-BY 4.0 International license.

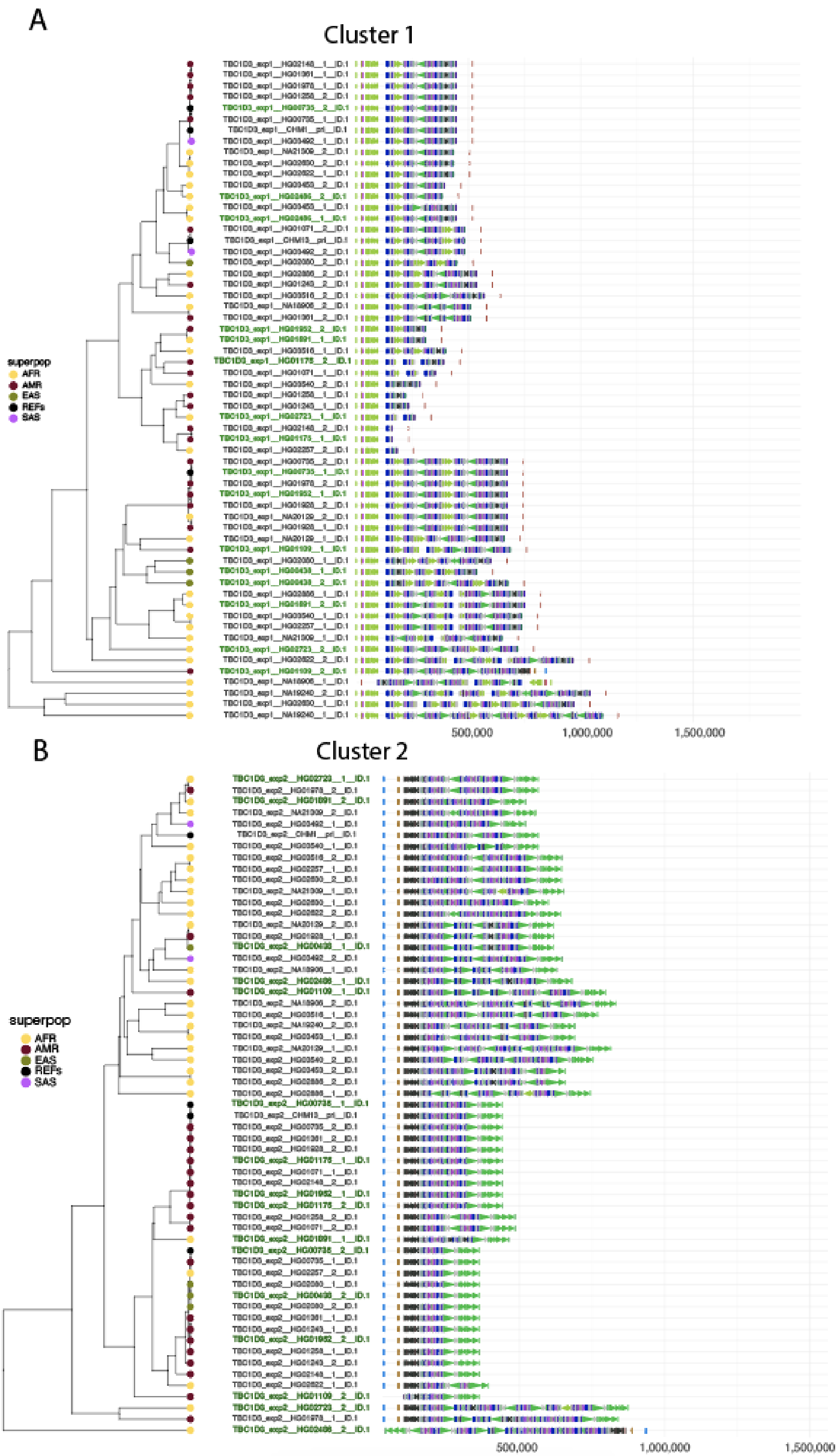

Supplementary Figure S2: UPGMA clustering of *TBC1D3*. We hierarchically clustered validated assemblies based on duplicon content using UPGMA for cluster 1 **(A)** and 2 **(B)** (Methods). Superpopulations for each haplotype are included in the dendrogram, and assemblies rescued with Verkko are colored in green.

bioRxiv preprint doi: https://doi.org/10.1101/2024.03.12.584650; this version posted March 13, 2024. The copyright holder for this preprint (which was not certified by peer review) is the author/funder, who has granted bioRxiv a license to display the preprint in perpetuity. It is made available under aCC-BY 4.0 International license.

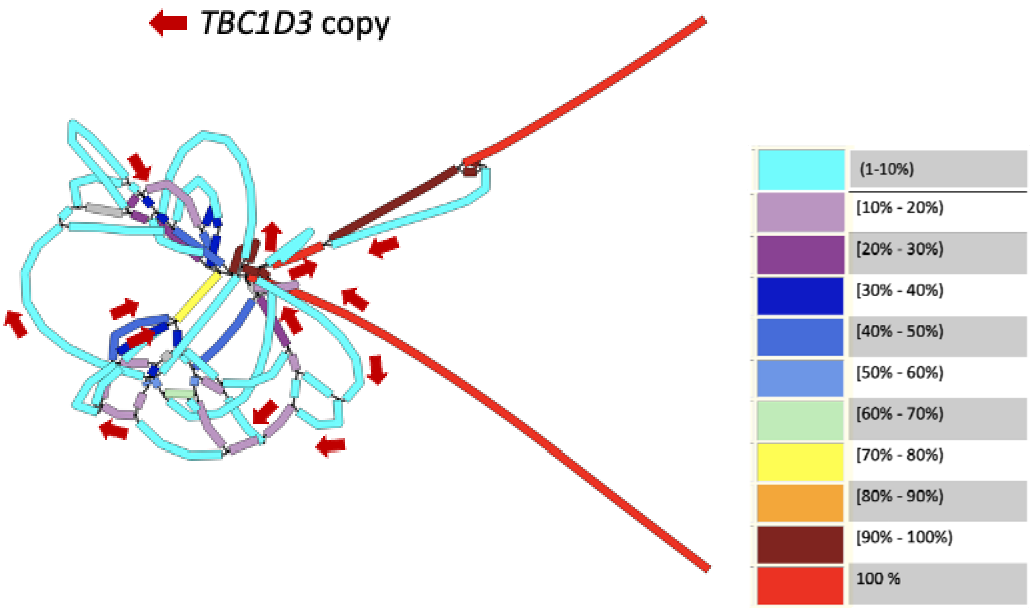

Supplementary Figure S3: Minigraph pangenome graph. We generated a pangenome graph for *TBC1D3* using validated human haplotypes with Minigraph (settings -S -xggs -L 250 -r 100000). Graph segments are colored to represent the proportion of haplotypes that span the given segment, with light red indicating 100% representation and light blue indicating a single-haplotype traversal. *TBC1D3* paralogs are marked with arrows along the graph. We observe that *TBC1D3* structural variation is poorly reduced by Minigraph, where most copies reduce to nodes with single-haplotype support.

bioRxiv preprint doi: https://doi.org/10.1101/2024.03.12.584650; this version posted March 13, 2024. The copyright holder for this preprint (which was not certified by peer review) is the author/funder, who has granted bioRxiv a license to display the preprint in perpetuity. It is made available under aCC-BY 4.0 International license.

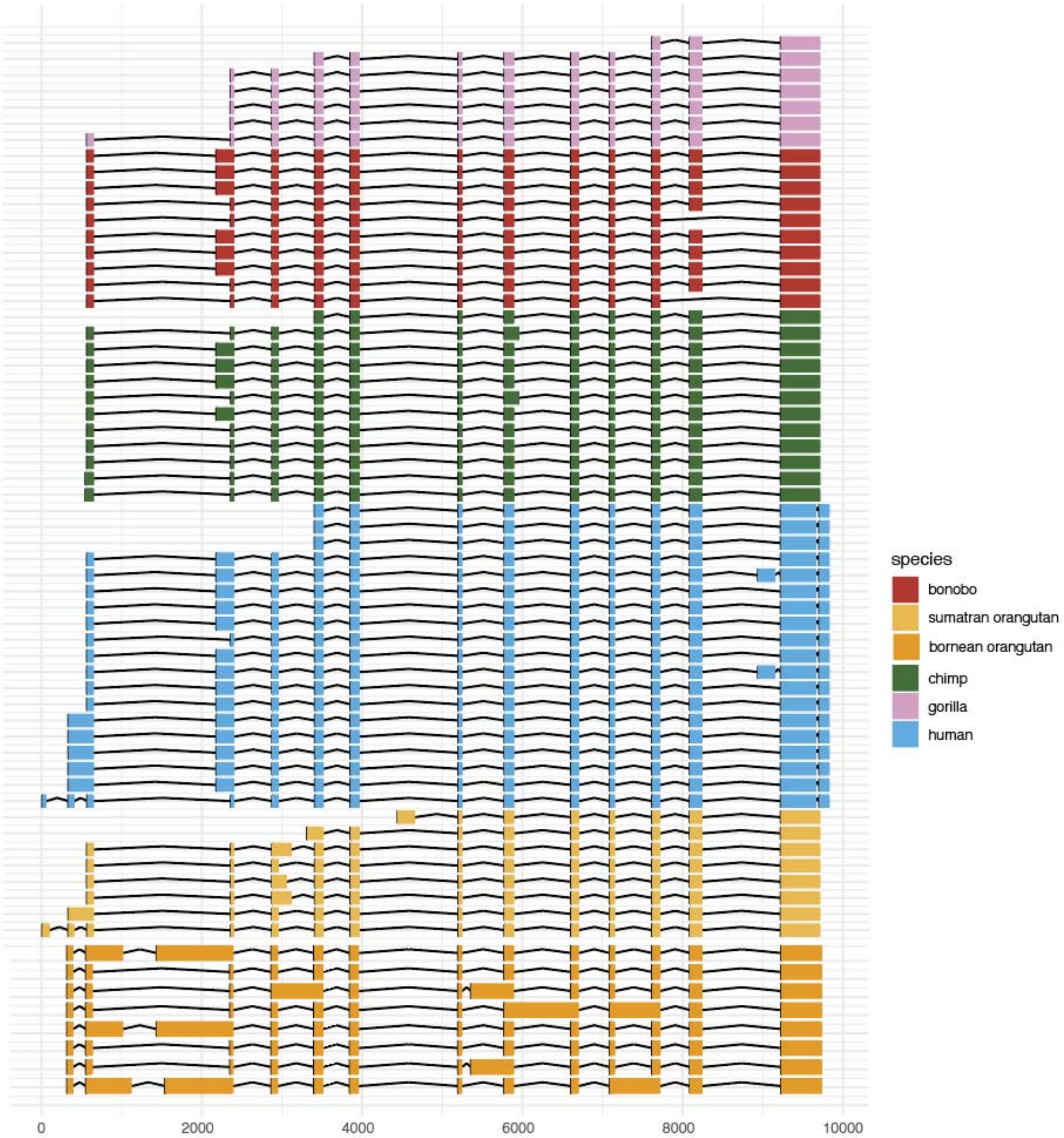

Supplementary Figure S4: Expressed *TBC1D3* paralog isoforms. Paralog-specific isoforms were selected for each primate based on their length, mapping quality, and expression support. We observe expression of *TBC1D3* from all ape lineages examined; however, for ORF analysis Bornean orangutan and gorilla isoforms were removed due to lack of expression support or intron retention.

bioRxiv preprint doi: https://doi.org/10.1101/2024.03.12.584650; this version posted March 13, 2024. The copyright holder for this preprint (which was not certified by peer review) is the author/funder, who has granted bioRxiv a license to display the preprint in perpetuity. It is made available under aCC-BY 4.0 International license.

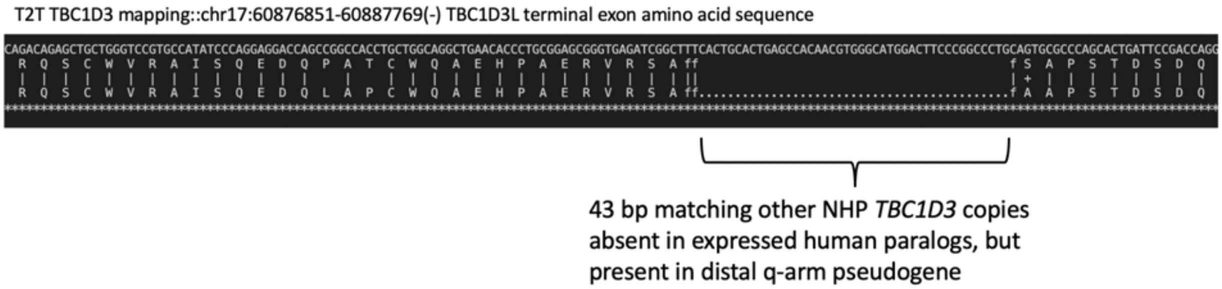

Supplementary Figure S5: Missing 43 bp deletion in human distal *TBC1D3* pseudogenes. Human-expressed *TBC1D3*, with ORF modified by a 43 bp deletion, was mapped to all primate *TBC1D3* copies, including all human *TBC1D3* copies, with prosplign (Kiriyutin et al. 2017). Prosplign predicts the genomic nucleotides that represent codons of a given amino acid. We observe that distal *TBC1D3* pseudogenes lack the 43 bp deletion universally present in cluster 1 and 2 human *TBC1D3* copies.

bioRxiv preprint doi: https://doi.org/10.1101/2024.03.12.584650; this version posted March 13, 2024. The copyright holder for this preprint (which was not certified by peer review) is the author/funder, who has granted bioRxiv a license to display the preprint in perpetuity. It is made available under aCC-BY 4.0 International license.

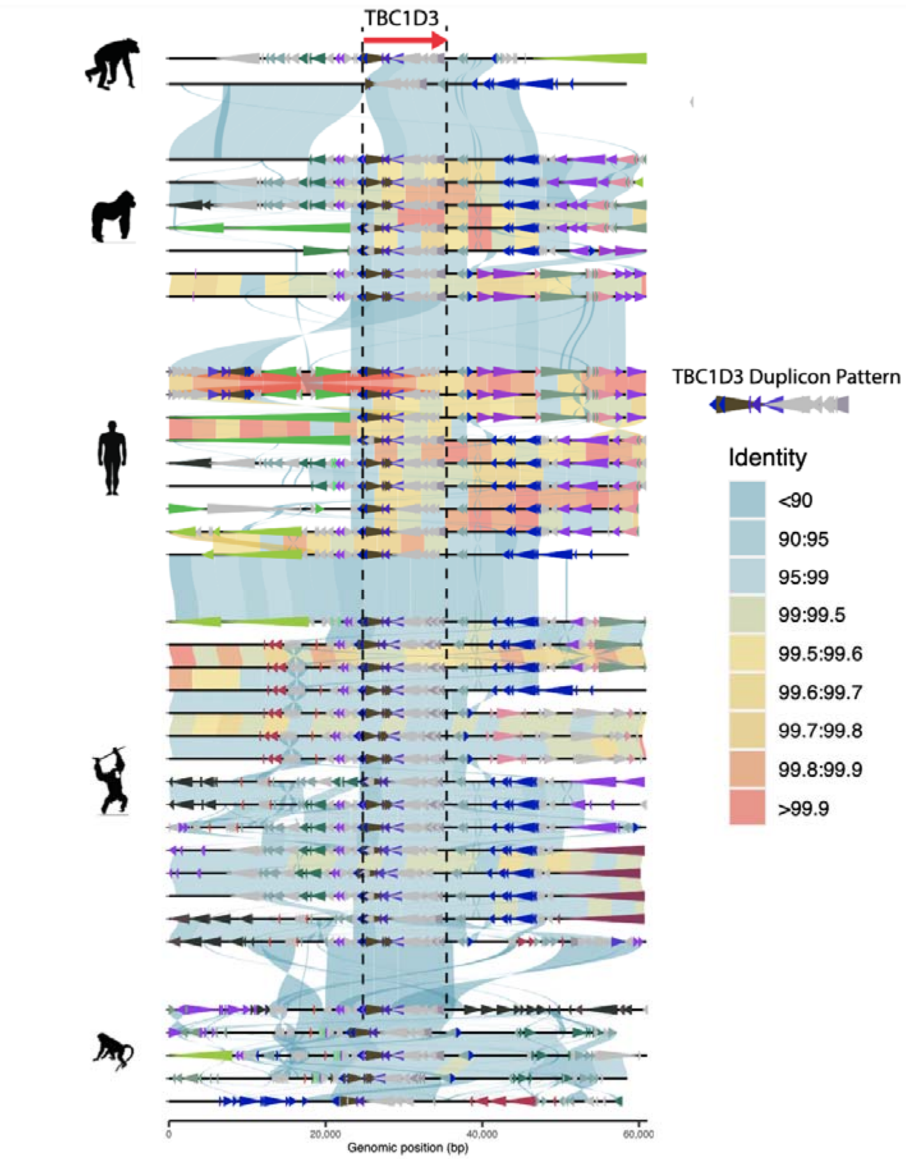

Supplementary Figure S6: Local *TBC1D3* duplicon structure supports independent expansion. *TBC1D3* copies from clusters 1 and 2, along with 25 kbp flanking sequence, were extracted and mapped to one another. We observe that these paralogs consistently map best, with highest sequence identity and contiguity, to paralogs from their same species of origin, consistent with the hypothesis of independent expansion. This contiguity is similarly observed in underlying duplicon content, shown with colored arrows and annotated with DupMasker.

bioRxiv preprint doi: https://doi.org/10.1101/2024.03.12.584650; this version posted March 13, 2024. The copyright holder for this preprint (which was not certified by peer review) is the author/funder, who has granted bioRxiv a license to display the preprint in perpetuity. It is made available under aCC-BY 4.0 International license.

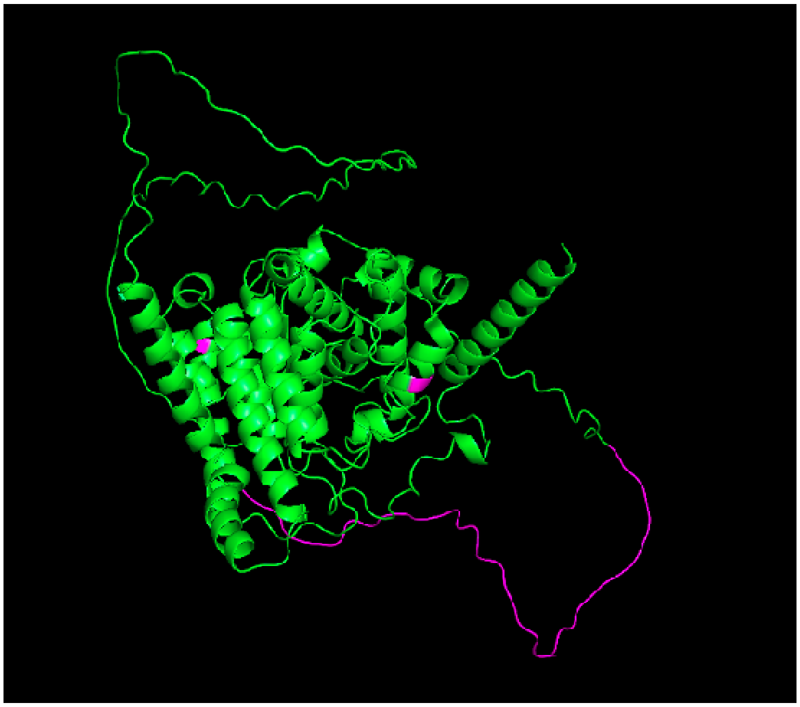

Supplementary Figure S7: Human *TBC1D3* predicted tertiary structure. Human *TBC1D3* was predicted with Alphafold2 (https://alphafold.ebi.ac.uk/). Human lineage amino acid changes, including the modified carboxy terminus, are indicated with violet. We observe that the 41 aa novel C-terminus tertiary structure could not be predicted and is disordered.

bioRxiv preprint doi: https://doi.org/10.1101/2024.03.12.584650; this version posted March 13, 2024. The copyright holder for this preprint (which was not certified by peer review) is the author/funder, who has granted bioRxiv a license to display the preprint in perpetuity. It is made available under aCC-BY 4.0 International license.

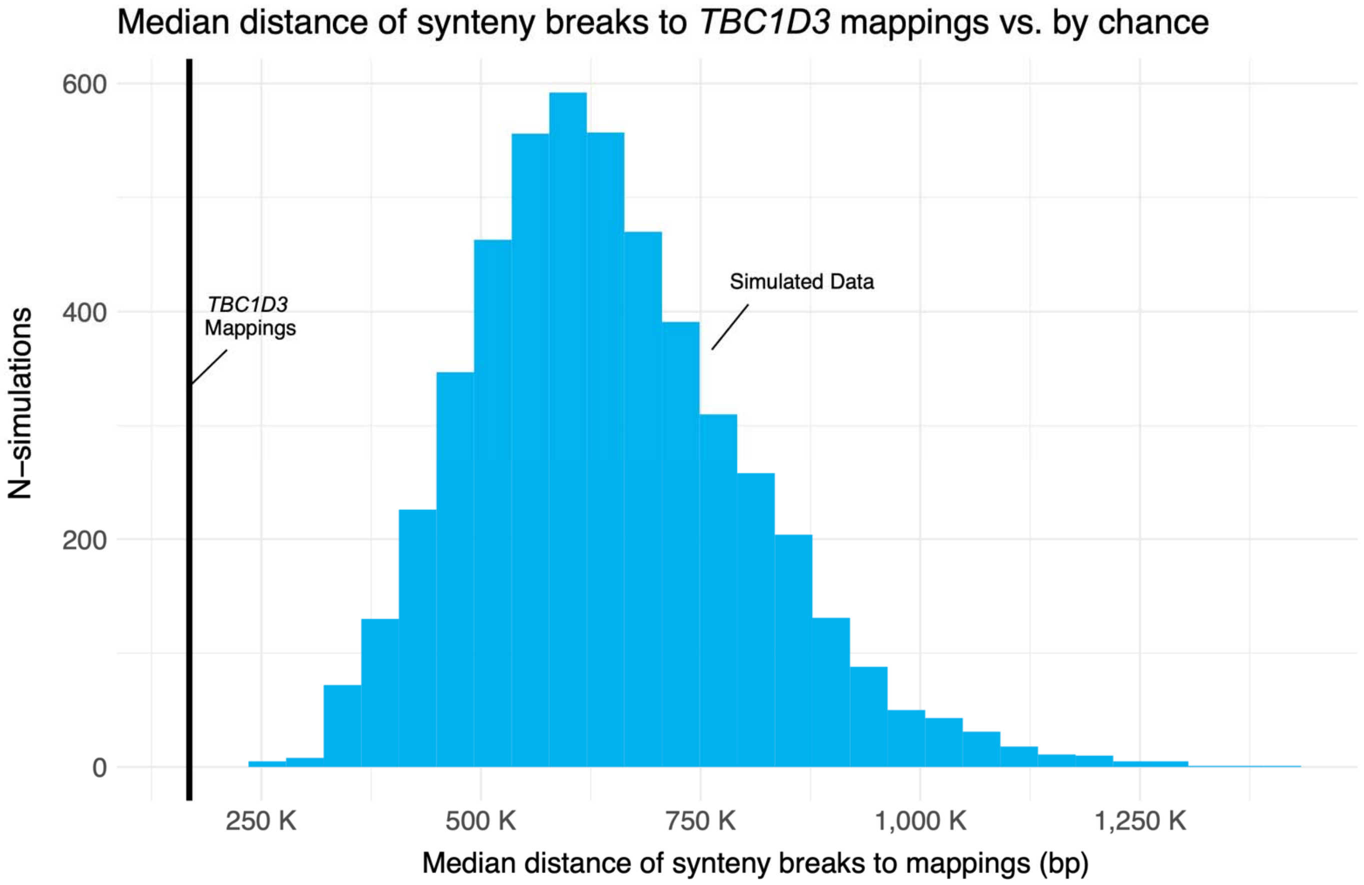

Supplementary Figure S8: *TBC1D3* vs. random genomic sequence permutation. Sequences of 11 kbp were randomly selected from orthologous primate chromosome 17 contigs at the same quantity as the observed *TBC1D3* copies contained within the chromosome. We calculated the median distance of this sampling and repeated this experiment in 5000 permutations, comparing median distance relative to true *TBC1D3* mappings, marked in black.

bioRxiv preprint doi: https://doi.org/10.1101/2024.03.12.584650; this version posted March 13, 2024. The copyright holder for this preprint (which was not certified by peer review) is the author/funder, who has granted bioRxiv a license to display the preprint in perpetuity. It is made available under aCC-BY 4.0 International license.

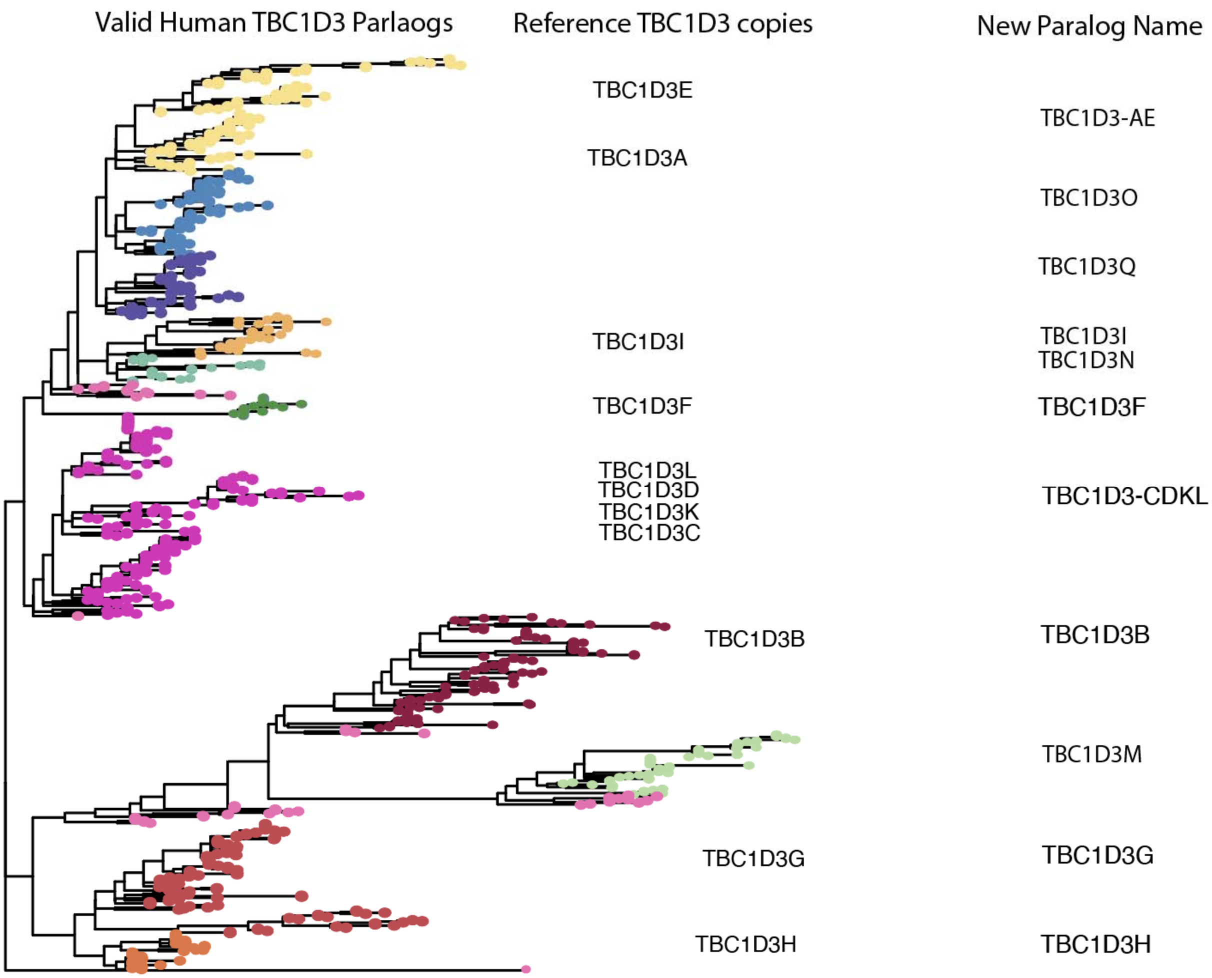

Supplementary Figure S9: Pangenomic clustering and naming. Maximum likelihood phylogeny with ~9600 bp of all human *TBC1D3* cluster 1 and 2 copies, outgrouped to chimpanzee *TBC1D3*. The paralog groups are defined by a heuristic intra-group allelic cutoff based on expected allelic variation in SD sequence (Methods). The first column of labels shows reference GRCh28 paralog locations within the phylogeny. The final column shows the new name given to the associated clusters. Most inherited the name assigned in GRCh28, or a concatenation when multiple paralogs mapped to a common cluster (*TBC1D3-AE; TBC1D3-CDKL*). Four novel population paralogs not included in GRCh38 were identified (*TBC1D3-M*,*TBC1D3-N*, *TBC1D3-O*, *TBC1D3-Q*).

bioRxiv preprint doi: https://doi.org/10.1101/2024.03.12.584650; this version posted March 13, 2024. The copyright holder for this preprint (which was not certified by peer review) is the author/funder, who has granted bioRxiv a license to display the preprint in perpetuity. It is made available under aCC-BY 4.0 International license.

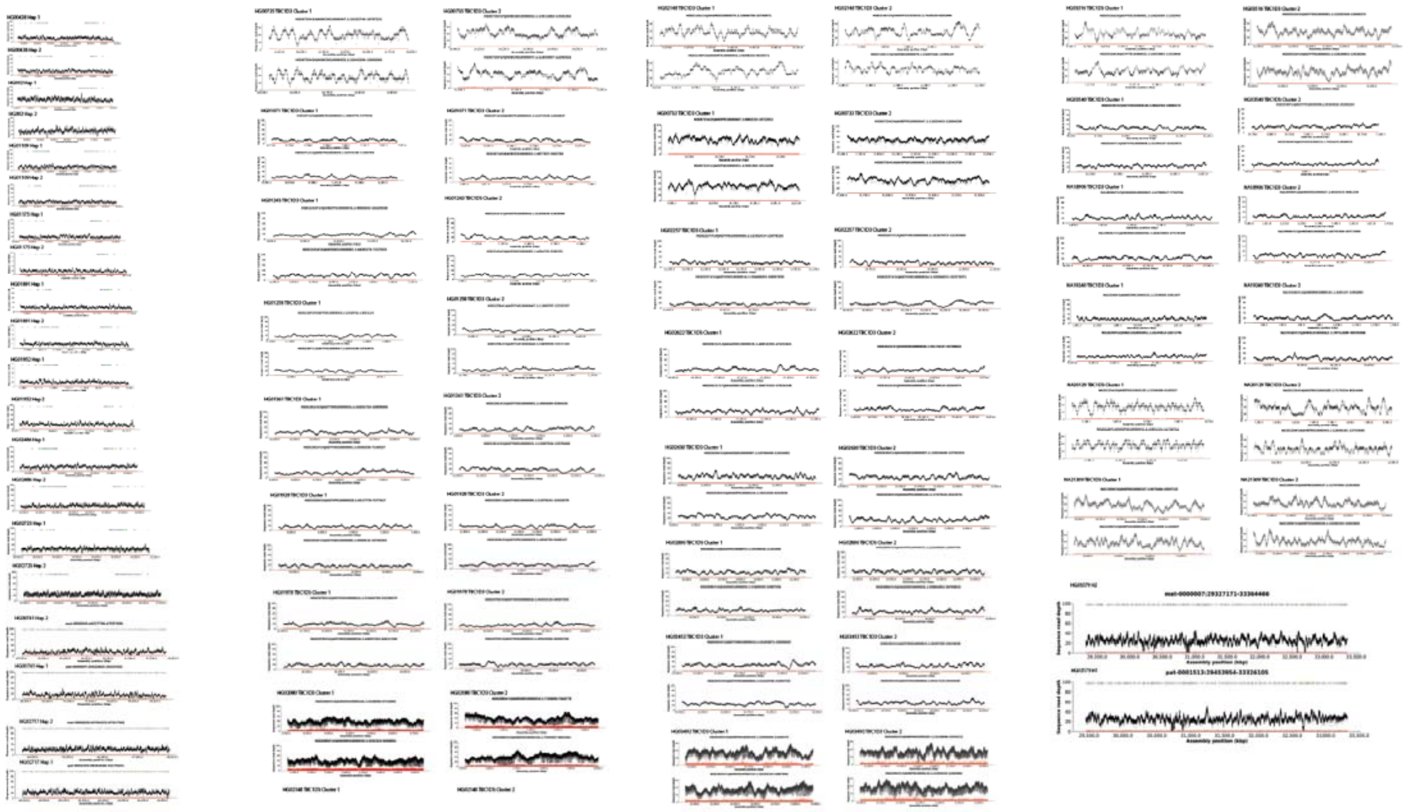

Supplementary Figure S10: NucFreq validation. The haplotypes' assembly accuracy was tested with NucFreq. Here, accuracy over each haplotype of *TBC1D3* cluster 1 and cluster 2 is demonstrated. Endogenous HiFi reads used to assemble respective phylogenies were mapped back onto the respective assemblies. We illustrated primary and secondary most prevalent base calls for reads aligned over a portion of an assembly in black and red, respectively. We expect primary base-call coverage to be relatively consistent, and consistently higher than the secondary base call, which should illustrate the sparse inaccuracies of HiFi sequencing.

SUPPLEMENTARY TABLES

Supplementary Table S1: Primate *TBC1D3* copy number.

Species	Scientific Name	Number of *TBC1D3* loci per haplotype |	Number of *TBC1D3* genes (Hap1 | Hap2)
Human ***	*Homo sapiens*	4 | 4	11 | 22
Gorilla*	*Gorilla gorilla*	4 | 4	13 | 11
Chimpanzee*	*Pan troglodytes*	4 | 4	8 | 8
Bonobo*	*Pan paniscus*	4 | 4	9 | 9
Sumatran orangutan*	*Pongo abelii*	8 | 9	23 | 23
Bornean orangutan*	*Pongo pygmaeus*	5 | 7	17 | 21
Gibbon*	*Symphalangus syndactylus*	11 | 11	31 | 30
Macaque**	*Macaca mulatta*	4 | 4	29 | 20

bioRxiv preprint doi: https://doi.org/10.1101/2024.03.12.584650; this version posted March 13, 2024. The copyright holder for this preprint (which was not certified by peer review) is the author/funder, who has granted bioRxiv a license to display the preprint in perpetuity. It is made available under aCC-BY 4.0 International license.

Gelada	*Theropithecus gelada*	3 | 3	31 | 24
Owl monkey**	*Aotus nancymaae*	4 | 4	8 | 8
Marmoset**	*Callithrix jacchus*	2 | 2	2 | 2
Mouse Lemur	*Microcebus murinus*	0 | 0	0 | 0

*Genomes sequenced as part of the Primate T2T Consortium (Makova et al. 2023). **Genomes sequenced and assembled as part of Mao et al. 2024. ***Genomes sequenced as part of human T2T project and Human Pangenome Reference Consortium (Nurk et al. 2022; Liao et al. 2023).

bioRxiv preprint doi: https://doi.org/10.1101/2024.03.12.584650; this version posted March 13, 2024. The copyright holder for this preprint (which was not certified by peer review) is the author/funder, who has granted bioRxiv a license to display the preprint in perpetuity. It is made available under aCC-BY 4.0 International license.

Supplementary Table S2: Primate genome assembly statistics.

Species	Scientific Name	HiFi Coverage	ONT Coverage	Assembly Method	Assembly N50 (Hap1 | Hap2) (Mbp)
Gelada	*Theropithecus gelada*	50.24	NA	Hifiasm 0.15.2	109.0 | 93.6
Mouse Lemur	*Microcebus murinus*	30.25	NA	Hifiasm 0.15.2	29.4 | 26.7
Human	*Homo sapiens*	30	120	NA	150.62
Gorilla*	*Gorilla gorilla*	107	165	Verkko 1.4	151.43 | 150.80
Chimpanzee*	*Pan troglodytes*	69	127	Verkko 1.4	147.43 | 140.84
Bonobo*	*Pan paniscus*	131	169	Verkko 1.4	147.03 | 147.48
Sumatran orangutan*	*Pongo abelii*	102	91.9	Verkko 1.4	143.46 | 140.60
Bornean orangutan*	*Pongo pygmaeus*	62.6	200	Verkko 1.4	139.77 | 139.21
Gibbon*	*Symphalangus syndactylus*	83.2	111	Verkko 1.4	144.67 | 145.50
Macaque**	*Macaca mulatta*	38.91	NA	Hifiasm 0.15.2	18.81 | 19.01
Owl Monkey**	*Aotus nancymaae*	36.57	NA	Hifiasm 0.15.2	55.92 | 44.99
Marmoset**	*Callithrix jacchus*	39.08	NA	Hifiasm 0.15.2	103.97 | 87.06

*Genomes sequenced as part of the Primate T2T Consortium (Makova et al. 2023). **Genomes sequenced and assembled as part of Mao et al. 2024.

Supplementary Table S3: Summary of Iso-Seq FLNC sequencing data.

Sample	***TBC1D3* reference copies**	Total number of sequencing reads	Reads mapped to ***TBC1D3***
Human fetal brain	14	2.33E+05	33138
Human iPSCs**	14	2.64E+08	11366
Gorilla testis*	15	4.68E+06	557
Chimpanzee testis*	8	3.00E+06	1461
Bonobo testis*	9	2.23E+06	915
Sumatran orangutan testis*	28	3.11E+06	2032
Bornean orangutan testis*	20	4.20E+06	6836

*Iso-Seq FLNC data generated as part of Makova et al. 2023. **Human iPSCs generated as part of Cheung et al. 2023.

Supplementary Tables S4-S10

Provided in a separate Excel file.
